# Spatial Distribution, Contamination Assessment and Origin of Soil Heavy Metals in the Danjiangkou Reservoir, China

**DOI:** 10.3390/ijerph20043443

**Published:** 2023-02-15

**Authors:** Qiuyao Dong, Chao Song, Dongxue Yang, Yuqing Zhao, Mingjiang Yan

**Affiliations:** 1 Institute of Hydrogeology and Environmental Geology, Chinese Academy of Geological Sciences, Shijiazhuang 050061, China; 2 Key Laboratory of Quaternary Chronology and Hydro-Environmental Evolution, Chinese Geological Survey, Shijiazhuang 050061, China; 3 Institute of Earth Sciences, China University of Geosciences (Beijing), Beijing 100083, China

**Keywords:** topsoil, heavy metal, contamination assessment, spatial distribution, origin

## Abstract

Soil heavy metal contamination is crucial due to menacing food safety and mortal health. At present, with the fast advancement of urbanization and industrialization, heavy metals are increasingly released into the soil by anthropogenic activities, and the soil ecosystem contamination around the Danjiangkou Reservoir is directly associated with water quality security of the reservoir. In this paper, using 639 soil samples from the Danjiangkou Reservoir, Henan Province, China, we studied a variety of space distribution characteristics of heavy metals in soil. Geographic information system analysis (GIS), geo-accumulation index (I_geo_), contamination factor (CF), principal component analysis (PCA) model, and positive matrix factorization (PMF) model were used together to recognize and quantify the distribution, contamination, and origin of heavy metals. We uncovered an exceptional variety of heavy metal concentrations among the tested soils: the mean arsenic (As), cadmium (Cd), cobalt (Co), chromium (Cr), manganese (Mn), nickel (Ni), zinc (Zn), lead (Pb) and mercury (Hg) concentrations (14.54, 0.21, 18.69, 81.69, 898.42, 39.37, 79.50, 28.11, 0.04 mg/kg, respectively, in the topsoil (0–20 cm depth)), all exceed their background values. The mean I_geo_ value and CF values of these trace elements are both in descending order: Cd > Co > Mn > Ni > Pb > Zn > Cr > As > Hg. Cd was the highest contributor to the assessment of heavy metal pollution, with an average I_geo_ value over three, indicating that the study area is modestly contaminated by Cd. The PCA analysis and PMF model revealed three potential sources, including natural sources (PC1) for Cr, Co, Mn and Ni; agricultural sources (PC2) for Cd, Zn and Hg; and industrial emissions and transportation sources (PC3) for Pb. This study displays a map of heavy metal contamination in the eastern area topsoil of the Danjiangkou Reservoir, showing the most severe pollutant is Cd, which poses a threat to the water quality security of Danjiangkou Reservoir and provides a significant source identification for future contamination control.

## 1. Introduction

Soil is key to agricultural activity, human survival and ecosystem balance [1,2]. Currently, with the headway of urbanization, farming and industrialization, anthropogenic impacts on the soil are intensifying, releasing more and more heavy metals into the soil, and heavy metal contamination is having an increasing impact on soil ecosystems [3,4,5]. Soil heavy metals are exceedingly poisonous, long-lasting, collect effectively in crops and are troublesome to biodegrade [6]. Once heavy metals contaminate the soil, they accumulate through trophic levels rather than easily decompose in the biological material cycle, causing irreversible and long-term harm to ecosystems and human health due to their ubiquity, bioavailability, toxicity and durability. In recent years, the contamination of soil by heavy metals has drawn incredible consideration from both analysts and environmental supervisors [7,8,9].

Extensive research shows that heavy metals in soils are broadly influenced by natural background levels and anthropogenic activities. Natural sources include mainly parent material and weathering of rocks [10,11]. Anthropogenic activities include industrial emissions [12], mining and smelting activities [13], fertilizers and agrochemical application [12], sewage irrigation [14], sludge application [15] and vehicle exhaust [16]. Subsequently, determining and quantifying the distribution, pollution and origins of heavy metals in soils are fundamental for territorial contamination control and administration [17,18]. As of late, the assessment records of soil heavy metals, involving single factor pollution index (PI), pollution factor (CF), potential ecological risk index (RI), geo-accumulation index (I_geo_) and so on, are normally utilized to assess the extent of the contamination of soil heavy metals [19,20,21]. Geostatistical strategies are utilized to analyze the spatial variability of heavy metals [22]. For example, geostatistical methods such as kriging interpolation and inverse distance weight interpolation (IDW) are usually utilized to analyze the spatial dissemination of heavy metals on diverse spatial scales [23,24]. Different methods containing multivariate statistical analysis, PCA and PMF are utilized for subjective or quantitative source recognizable proof. The PMF divides a sample into diverse variables and analyzes them [25]. The PMF has been prescribed by the US EPA for evaluating source apportionment and calculating the commitment of natural poisons. It is the foremost prevalent and successful strategy prescribed by the US EPA, and it is broadly utilized [26,27]. The combination of PCA and PMF could improve the accuracy of quantitative identification of soil heavy metal sources.

Several studies have reported that soil ecosystems around numerous reservoirs are highly affected by human activities, with complex pollution sources and multiple routes [28]. Danjiangkou Reservoir, as the largest man-made freshwater lake in Asia, is one of the important pure-water sources for China’s Middle Route of South–North water diversion project. The water diversion project from South to North, moreover, could be a major vital infrastructure project in China [29]. It is concerned with the issue of water quality safety and the threat of soil quality degradation and soil pollution within the reservoir ecosystem. The contamination of soil heavy metal is herein deemed to be one of the greatest threats. However, there are few studies on the characteristics and contamination evaluation of soil trace elements around the reservoir region. Therefore, to investigate the impacts of different components on the content of trace elements in topsoil along the Danjiangkou Reservoir, to determine the ecosystem dangers of soil heavy metals contamination to the reservoir, and to provide scientific ways for ecological reconstruction of the reservoir area, the purpose of this paper is to: (1) analyze the spatial pattern and the trend of change of heavy metals in soil in the eastern area of the Danjiangkou Reservoir, Henan Province, China; (2) assess heavy metal contamination from As, Cd, Co, Cr, Mn, Ni, Zn, Pb, and Hg occurring in soil; and (3) identify potential origins of trace elements and evaluate their relative contribution of pollutants.

## 2. Materials and Methods

### 2.1. Description of the Study Area

**Figure 1:**
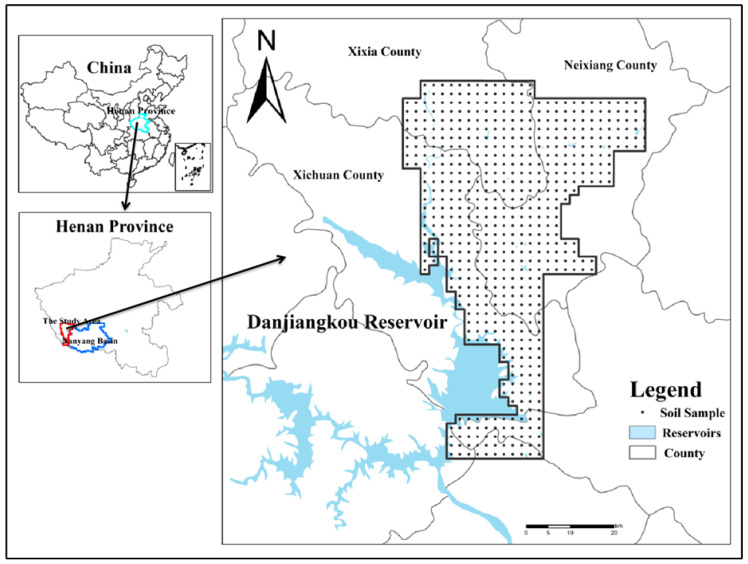
Distribution of sampling sites in the study area.

The study area (longitude: 111°24′~112°00′, latitude: 32°34′~33°21′) is mainly situated in the southwest of Nanyang City, Henan Province, with Nanyang Basin in the west and the Qinling branch of Funiu Mountain foothills in the north, and the Danjiangkou Reservoir, which is a significant water resource for more than 20 large and medium-sized cities along the route, including Beijing, Tianjin and Shijiazhuang of China, in the south. This area mainly consists of Xixi County, Neixiang County and Xixia County, and totals approximately 2490 km^2^ (Figure 1). The topographic trend of higher elevation within the northwest and lower elevation within the southeast is because the study area is situated on the eastern extension of the Qinling Mountains. Hills and mountains constitute the main types of landforms in this area [30]. It is a subtropical monsoon climate that is relatively arid and has an annual mean temperature of around 15.3 °C. The agrotypes in the study area are primarily yellow-brown soil, brown soil and fluvo-aquic soil with some paddy soil. The land use pattern is mainly arable land, grassland and other land, and cultivated land accounts for more than 80% of the full area. At the same time, several types of metallic and non-metallic deposits have been discovered in the study area, and the deposits are characterized by large reserves, wide distribution, stable levels and easy mining. According to the National Mineral Resources Database 2022, there are about 123 mineral occurrences in the study area. Rapid and intensive agricultural production in this study area, such as fertilizer, animal excrement and pesticide application, and industrial production related to steel, metal and press, may make the number of trace elements within the soil worse. In summary, the special surroundings (background, topography, agrotype, land use pattern, ore resources and other factors) affect the content and the spatial distribution patterns of heavy metals.

### 2.2. Collecting Soil Samples and Physico-Chemical Property Analysis

This study is based on the results of the “1:250,000 Land Quality Geochemical Survey Project in Nanyang Basin (Nanzhao-Xichuan Area)”, which was carried out in the Nanzhao-Xichuan area from 2019 to 2022. There were 639 topsoil (0–20 cm) samples in total. The geolocation of sampling sites was tracked via GPS. Every analytical sample is composed of four samples from an adjacent 1 km^2^ area. Within the same period, the altitude, land use, crop species, longitude and latitude of sampling points were noted, and rocks, insects and other debris in the soil were removed and put into cloth bags. All the tests were air-dried at room temperature, passed through 2 mm nylon sifters, and after that put away in hermetically fixed polyethylene packs for chemical examination [31].

The sample analysis and testing work for this project was tested by Henan Province Rock and Mineral Testing Center and North China Nonferrous Metals Yanjiao Center Laboratory Company Limited. Laboratory and tests have been analyzed. The testing of samples was processed with concentrated acid (HNO_3_-HF-HClO_4_) [32] and the concentrations of Cd, Co, Ni and Pd were decided utilizing Inductively Coupled Plasma Mass Spectrometry (ICP-MS, NexION 300Q, Perkin Elmer, MA, USA). The samples were digested with a concentrated acid (HNO_3_-HC-HF-HClO4) and the contents of Cr, Mn and Zn were determined utilizing an Inductively Coupled Plasma Optical Emission Spectrometer (ICP-OES, Optima 7300DV, Perkin Elmer, MA, USA) [33]. As and Hg were digested with a concentrated acid (HNO_3_ and HCl) and their contents were determined using Atomic Fluorescence Spectrometer (AFS, SK-2003A, Beijing, China) [34]. For quality control (QC) and quality assurance (QA), clear control, copy tests and standard reference soils (GBW07419; Center for Certified Reference Materials, Beijing, China) were utilized [7]. The detection limits of each analytical method are shown in Table 1. The amounts measured for the standard reference soils were inside stability ranges of the certified values.

### 2.3. Assessment of Heavy Metal Pollution

#### 2.3.1. Contamination Factor (CF)

The values of the CF index were obtained by isolating the concentration of each heavy metal by the foundation concentrations [35]. The $C_{f}$ is calculated as follows:

(1) $$C_{f}=\frac{C_{m\;sample}\;}{C_{m\;backgroud}\;},$$

where ‘$C_{m\;sample}$’ refers to the concentration value of an index and ‘$C_{m\;backgroud}$’ is the metal content from a natural reference, such as those reported in Hans Wedepohl [36]. When $C_{f}$ < 1, it is described as low pollution level; 1 ≤ $C_{f}$ < 3 may be a moderate pollution level; 3 ≤ $C_{f}$ < 6 is a considerable pollution level, and $C_{f}$ ≥ 6 could be an exceptionally high pollution level.

#### 2.3.2. Geo-Accumulation Index (I_geo_)

The I_geo_ was used to decide the extent of trace element contamination in the soil tests. This assurance equation was presented by Müller [37] as a normal quantitative estimation of the concentration of contamination in soils or aquatic sediments. The condition utilized for the calculation of I_geo_ was:

(2) $$I_{\mathrm{geo}}=\log_{2}\frac{C_{n}}{K*B_{n}},$$

where $C_{n}$ is the content of metals in the soil; $B_{n}$ is the background value and K = 1.5 is the background matrix correction factor. Based on the $I_{\mathrm{geo}}$ values, the contamination could be classified into seven categories: practically uncontaminated, uncontaminated to moderately contaminated, moderately contaminated, moderately to strongly contaminated, strongly contaminated, strongly to extremely contaminated, and extremely contaminated when the $I_{\mathrm{geo}}$ is <0, 0–1, 1–2, 2–3, 3–4, 4–5 and >5. A translation of the values obtained from this record is shown in Table 2.

#### 2.3.3. Positive Matrix Factorization (PMF) Model

The PMF, a method suggested by the U.S. Environmental Protection Agency (EPA), could demonstrate, based on PCA, the number of origins and the contribution of each origin based on the origins’ profile [38]. It offers a remarkable advantage for data preprocessing, determining the values of missing data in datasets, unpredictability estimation, and data plausibility analysis [39,40]. In this study, EPA PMF 5.0 was utilized to recognize and measure the soil samples from the diverse origins. The PMF breaks down the sample information matrix $x_{ij}$ into the origins profile matrix $h_{\mathrm{mj}}$ and a source contribution matrix $g_{\mathrm{im}}$:

(3) $$x_{ij}=\sum_{m=1}^{t}\left(g_{\mathrm{im}}h_{\mathrm{mj}}+e_{\mathrm{ij}}\right),$$

where i is the number of samples, *j* is the heavy metal types, t is the number of sources, and $e_{\mathrm{ij}}$ is the error for each sample.

In PMF analysis, the content and uncertainty of the sample need to be determined. The uncertainty ($u_{ij}$) is calculated as follows:

(4) $$x_{ij}\leq MDL,\;u_{ij}=\frac{5}{6}\times MDL,$$

(5) $$x_{ij}>MDL,\;u_{ij}=\sqrt{{\left(errorfraction\times x_{ij}\right)}^{2}+MDL^{2},}$$

where MDL is the species-particular methodology detection limit, and the errorfraction is a percentage of the measurement uncertainty.

The point of the PMF is obtained through minimizing the object function Q:

(6) $$Q=\sum_{i=1}^{p}\sum_{j=1}^{q}{\left[\frac{x_{\mathrm{ij}}-\sum_{m=1}^{t}g_{\mathrm{ij}}h_{\mathrm{ij}}}{u_{\mathrm{ij}}}\right]}^{2},$$

where $x_{\mathrm{ij}}$ is the heavy metal content, $u_{\mathrm{ij}}$ is the uncertainty of samples, p is the number of samples, q is the heavy metal type, and t is the number of sources.

### 2.4. Data Analysis

Geochemical maps and multivariate statistical analysis are effective tools for identifying pollution sources. The chart of the spatial interpolation of the nine topsoil heavy metals and properties were drawn utilizing ArcGIS 10.6 (ESRI, Redlands, CA, USA) computer program utilizing the inverse distance weighting (IDW) method. A cartogram of the PCA of the soil’s heavy metal contents and the chemical and physical properties of soil were obtained using SPSS 20.1 (SPSS Inc., Chicago, IL, USA). At that point, information preprocessing, containing numerical and graphic information and box or line graphs of the heavy metals, was made using Microsoft Excel 2019 and Origin 10.0. Based on the understanding of the sources of heavy metals in soil, the contribution of different contamination sources of soil heavy metals was quantitatively analyzed using EPA PMF 5.0.

## 3. Results and Discussion

### 3.1. The Descriptive Statistical Parameters of Heavy Metal Concentrations in Topsoil

Analysis of heavy metal concentration in the soil can reveal the degree of pollution. The descriptive characteristics of heavy metals in the topsoil are listed in Table 3. The descriptive data for nine soil heavy metals from 639 soil samples of the study area are displayed. The mean values of Cd (0.21 mg/kg), Co (18.69 mg/kg), Mn (898.41 mg/kg), Ni (39.37 mg/kg), Pd (28.11 mg/kg), Hg (0.04 mg/kg), and Zn (79.50 mg/kg) were beyond the median values. Only the average of As (14.45 mg/kg) and Cr (81.69 mg/kg) were below the median values. The mean of all nine heavy metals is larger than the background values (BV) of Henan Province of heavy metals in soil, which were obtained from [41], and the mean of Cd content was 3.04 times higher than the BV of Henan Province, reflecting a greater influence by anthropogenic activities. The standard deviations of Cr, Zn, and Mn were larger than that of other heavy metals, proposing a better degree of scattering [42]. A higher coefficient of variety (CV) shows more prominent irregularity and wide potential for anthropogenic impacts [43]. Concurring to Wilding [44], tall variety, moderate variety, and low variety are characterized by values of CV > 36%, 16% < CV < 36%, and CV < 16%, in sequence. Hg (113%) had the greatest variation. Cd (48%) was high variation. Both exhibit high variability, higher coefficients of variation, greater potential for discontinuity and widespread anthropogenic influence.

**Figure 2:**
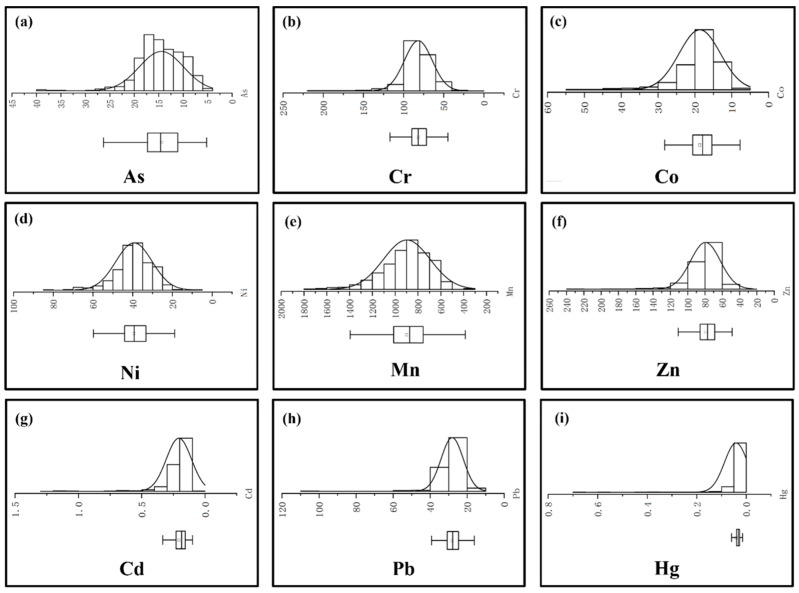
Histograms of heavy metal concentrations in topsoil for (**a**) As, (**b**) Cr, (**c**) Co, (**d**) Ni, (**e**) Mn, (**f**) Zn, (**g**) Cd, (**h**) Pb and (**i**) Hg.

In the absence of obvious human activities, the value of trace element content in regional soil usually follows normal distribution [45]. As shown in Figure 2, the soil heavy metals As, Co, Cr, Mn, Ni, and Zn are normally distributed. Heavy metals are not obviously affected by anthropogenic activities. However, there are many extremely high values of Cd, Pb, and Hg, a right deviation trend. Therefore, it is suggested that the abnormally high values of these three heavy metals are influenced by human activities, representing man-made imports of heavy metals to the soils. Therefore, it is believed that these three elements are affected by anthropogenic activities to some extent, and the abnormally high values may represent their accumulation and enrichment in the soil due to human emissions.

### 3.2. Space Distribution Pattern of Heavy Metals

**Figure 3:**
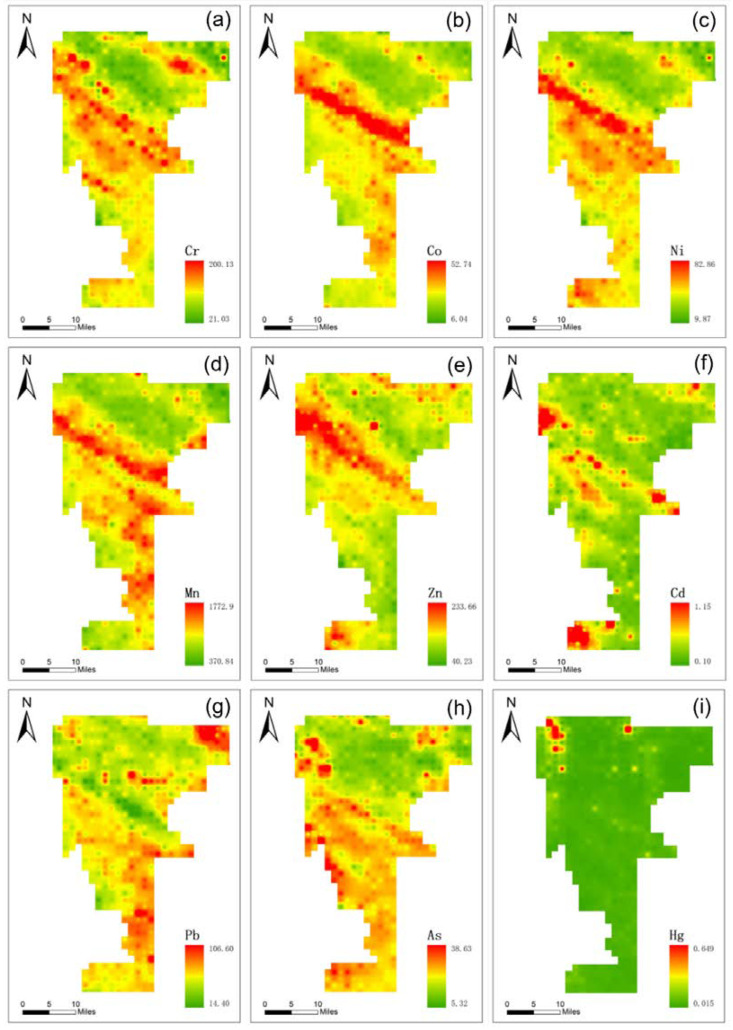
Spatial distributions in topsoil for (**a**) Cr, (**b**) Co, (**c**) Ni, (**d**) Mn, (**e**) Zn, (**f**) Cd, (**g**) Pb, (**h**) As and (**i**) Hg.

The spatial distribution pattern of soil trace elements is shown in Figure 3. In general, the spatial distribution pattern was clearly diverse from the north to the south of the study area. The concentrations of heavy metals were more prominent within the south than in the north. Laoguan River, a river of Han River system in Yangtze River Basin of China, drains into Danjiangkou Reservoir and passes through the northwest of the whole study area. Thus, the geomorphological features of the northwest portion of the study area are predominantly flooded river valley plains and erosion and denuded low hills. Soil with high porosity, large particles, poor organic matter concentrations and poor water and fertilizer conservation capacity, and the topsoil of the northeastern region of the study area have a low concentration of heavy metals. The high heavy metal hotspot areas are mainly distributed in the center of the south and southwest of the study area, whereas the low heavy metal hotspot areas are distributed in the northeast of the study area, with obvious scattered distribution. It appears that Mn, Cr, Co and Ni had a similar spatial distribution pattern. A few recent studies have detailed that Mn, Cr, Co and Ni in topsoil primarily started from the soil parent fabric [46]. Pb and As have similar spatial distribution characteristics and both are probably influenced by the same pollution sources. The high values are mainly distributed in the south-central region of the study area. Past studies found the most noteworthy concentrations of Pb and As within the soils encompassing industrial facilities and streets [47,48]. In comparison, the scattered dissemination within the high Cd region was more concentrated. The spatial distribution map of Cd, its high value area is roughly the same distribution as the agricultural production area, and its pollution source may come from agricultural pollution. Within the farmland soils, sources of contamination for the most part come from industrial production or other human activities. Subsequently, the areas with the greatest concentrations of soil heavy metals are those where anthropogenic activities are most intensive, especially industrial production and transportation.

### 3.3. Assessment of Heavy Metal Pollution

**Figure 4:**
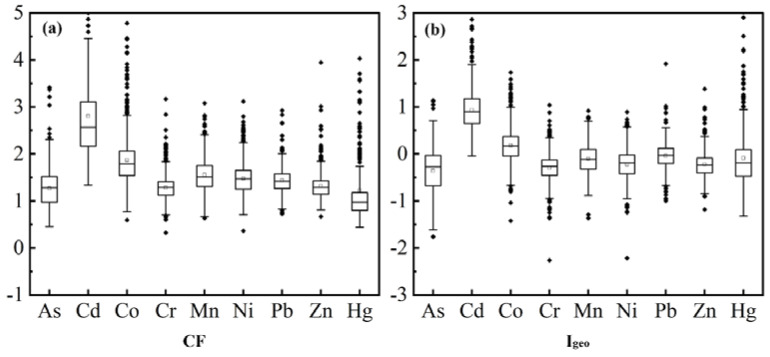
Boxplot of (**a**) contamination factor (CF) and (**b**) geo-accumulation index (I_geo_) of each metal in the study area.

For further research, to evaluate the degree of trace element contamination in the topsoil in the study area, indicators such as CF and I_geo_ were calculated (Figure 4). CF and I_geo_ are relatively well-established statistical methods and are commonly used for evaluating heavy metals pollution in soil. The mean CF values of soil heavy metals in order were Cd > Co > Mn > Ni > Pb > Zn > Cr > As > Hg. The mean CF values of the heavy metals in the study area ranged from 1 to 3, which is moderately heavy metal contamination (Figure 4a). This demonstrates that the pollution of soil heavy metals within the study zone comes from anthropogenic activities. The values of I_geo_ are shown in Figure 4 and demonstrate that the mean values of the I_geo_ of six heavy metals, As, Cr, Hg, Ni, Pb and Zn, were all less than 0, while the I_geo_ of Cd, Co and Mn were greater than 0. It can be seen that the topsoil in the study area was, overall, free of As, Cr, Hg, and Zn contamination; was slightly contaminated with Cd, Co, Mn, Ni, and Pb; and had localized medium-intensity contamination of Cd and Hg. The average I_geo_ values of heavy metals in topsoil were Cd (0.81) > Co (0.26) > Mn (0.01) > Ni (−0.06) > Pb (−0.09) > Zn (−0.22) > Cr (−0.26) > As (−0.31) > Hg (−0.54) in that order. Like the values of CF, Cd was the highest contributor to the appraisal of heavy metal pollution, with the mean value of I_geo_ above 3. Therefore, Cd was the foremost predominant contaminant in the topsoil of the study area.

### 3.4. Source Identification of Heavy Metals

#### 3.4.1. Source Identification Using PCA

**Figure 5:**
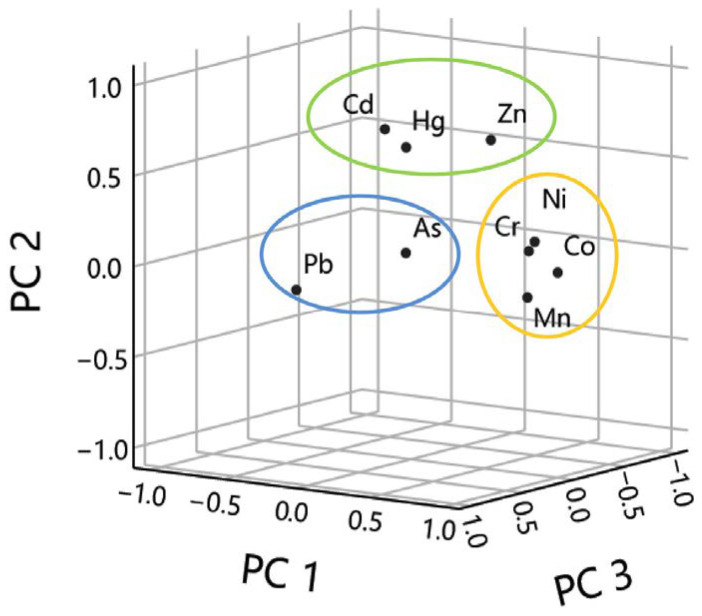
Contributions of sources to nine heavy metals based on PCA model.

PCA is a well-established and demonstrated statistical strategy routinely utilized to distinguish potential sources of heavy metals in topsoil [49,50]. In this study, heavy metal concentration information was analyzed by PCA, yielding three components based on varimax that in total contributed 69.6% of the change in topsoil contamination (Table 4 and Figure 5).

**Figure 6:**
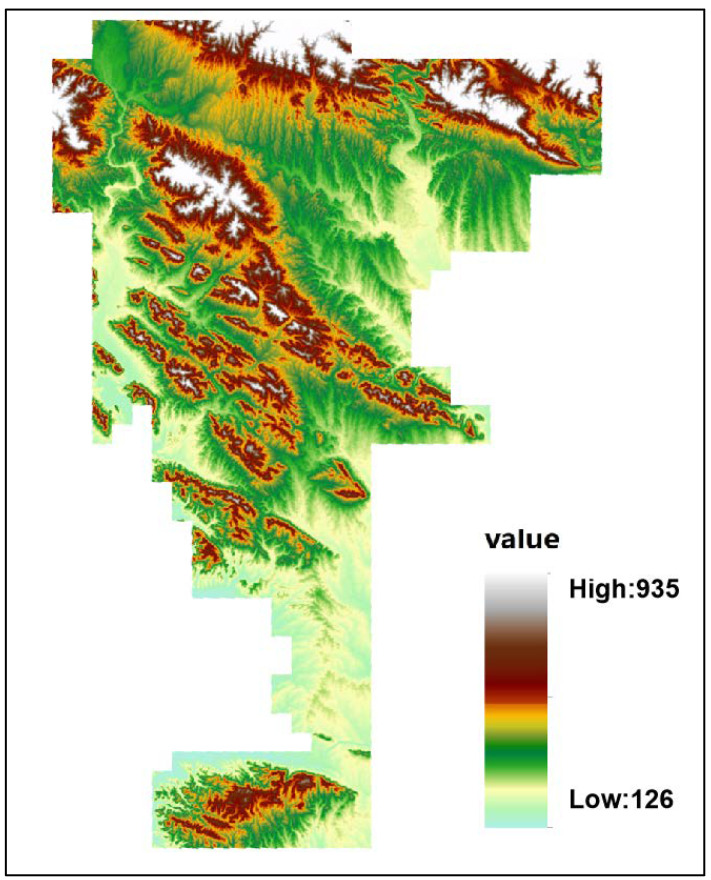
Digital elevation model (DEM) map of the study area.

PC1 contributed 40.9% of the fluctuation and included a strong positive stacking of Mn, Co, Ni and Cr (Figure 5), illustrating that those heavy metals appear to come from a comparable origin. The soil profile investigation for this seems clear. The CV values of Cr, Co, Ni, Mn and Zn contents were 21%, 30%, 23%, 23% and 22%, respectively, setting it within the low spatial variability. Most Cr aggregation in soils was close to the normal background levels that were controlled by the natural factors [48]. Thus, PC1 could be attributed to natural sources. The study area is located in the eastern section of the Qinling folded orogenic belt, with complex geology and wide stratigraphic distribution, mainly for the Paleozoic Qinling rock group. The rock types include gabbro, monazite, amphibolite, orthogabbro, granite porphyry and alkalic feldspar granite. The lithological effects are also attributed to natural sources. The lithological effects are also attributed to natural sources. Since the average contents of Co, Cr and Ni in the surface soil are close to the regional background values and they have similar spatial distribution characteristics with the DEM map (Figure 6), the geochemical characteristics of Co, Cr, Ni and Mn in the surface soil mainly depend on the soil parent material and natural soil formation process.

PC2, which accounts for 16.1% of this, add up to fluctuation and contain a solid positive stacking of Cd (77%), and a direct positive stacking of Pb, Zn and Hg (Figure 5). The high value area of cadmium is roughly the same distribution as the agricultural production area, and its pollution source may come from agricultural production. Previous studies found Cd is usually susceptible to soil entry by anthropogenic activities, mainly agricultural fertilizers, sewage discharge and industrial production [51]. The results of lasting (1989–2001) trials conducted at the Agricultural Experiment Station displayed that the contents of Pb, Zn and Cd within the soil indicated an increasing trend under different fertilization treatments [52]. The extensive use of phosphate and compound fertilizers has led to a persistent increment within the concentration of Cd in the soil [53,54,55]. Cd is additionally regularly utilized within the industrial production of various products, such as pigments, plastics and electroplating [56]. Thus, PC2 could be mainly attributed to the agricultural sources.

PC3 explained 12.61% of this total variance and includes a strong positive loading of Pb (69%), and a moderate positive loading of As (Figure 5). Earlier research has confirmed that the enrichment of Pb within the soil primarily related to vehicle exhaust [57]. This can be attributed to the fact that gasoline contains lead, which enters the air through vehicle exhaust emissions and eventually leads to particulate matter containing lead entering the soil through atmospheric deposition [58]. In spite of the fact that China prohibited the generation and selling of leaded gasoline in 2000, the substance of lead in soil is still a hot point in our society nowadays since lead incorporates a half-life of hundreds of years--a long time [59,60]. Pb is usually the signature trace element of motor vehicle pollution, and vehicle engines and tire wear and tear can emit large amounts of Pb into the environmental medium, accumulating into the soil through atmospheric deposition [61]. Metal smelters have a significant impact on the content of various trace elements in the atmosphere and soil and cause the increasing input of some heavy metals in the soil [62]. Thus, PC3 might be related to both industrial emissions and transportation sources.

#### 3.4.2. Quantitative Source Apportionment Using the PMF Model

**Figure 7:**
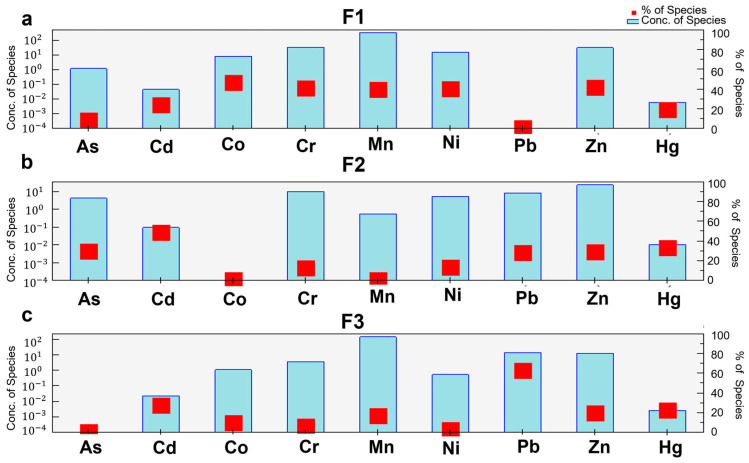
Source contribution of nine heavy metals for (**a**) F1, (**b**) F2 and (**c**) F3 based on PMF model.

In this query, we utilized PMF to measure the sources of poison in soil and contributions of trace elements and to superior partitioned trace elements from natural sources and the sources of human activities (Figure 7a). The PMF was utilized to quantitatively analyze the potential sources of heavy metals in the topsoil. After a few times of computer program debugging, to attain the objective of minimizing Q value, the optimal analytical outcomes about were obtained, and the factor number three was at long last decided as the optimal [63]. The values of the scaled residuals were from −3 to 3, and the fitting coefficients R2 between the measured and forecasted values of the trace elements were more noteworthy than 0.8. The strongest dependencies (R2 > 0.8) between the predicted and measured values were found for Pb and As, and the strong correlations (R2 > 7) were found for Co, Cr, Mn and Zn, suggesting that PMF can be well applied to prediction in this study. Thus, the PMF model could be effectively utilized for the assignment of soil heavy metal sources in this area [56].

Compared with other trace elements, the first source factor for Co (46%), Zn (41.2%), Cr (40.8%), Mn (39%) and Ni (40.3%) occupied a greater proportion, as shown in Figure 7a. The natural sources are the primary reason that restricts the distribution of these soil trace elements. Therefore, Factor 1 is considered as the natural source. The second source figure is related with moderately rich contents of Cd (Figure 7b), and source contribution of Cd reached 48.9%. Previous studies have shown that the study area has been affected by broadly active agrarian activities, counting the application of chemical fertilizers and sewage water system over the past few years [64]. These results demonstrated that the human activities sources are the major sources of Cd contamination, which is close to the results from PCA. Therefore, Cd originated mainly from the source of agricultural production [65,66,67]. For the third contamination source (Figure 7c), the weight value of Pb (62.9%) was more than that of other trace elements. High concentrations of lead in topsoil close most industrial foundations and along streets are comparative to past studies, proposing that lead in topsoil may too be related with industrial production and transportation sources [47,68,69].

The PMF analysis results are consistent with the PCA model results. The three potential sources in topsoil are the natural sources, the agricultural sources, and the mixture of industrial production and transportation sources.

## 4. Conclusions

Based on the analysis of 639 topsoil samples and nine heavy metals in this study area, the spatial distribution maps, multivariate statistics and receptor model were utilized to elucidate the content and spatial distribution features of heavy metals to investigate the sources of heavy metals in soil and calculate the relative contribution of various potential sources. In conclusion, the mean values for As, Cd, Co, Cr, Mn, Ni, Zn, Pb and Hg surpassed the corresponding Henan background values in the study area around the Danjiangkou Reservoir in Henan Province, China, which showed that the soil might be influenced by human activities. The concentrations of Co, Cr and Ni in topsoil were low, which mainly came from natural weathering. The high value regions of Cd, Zn and As were primarily influenced by agricultural production, and the contribution of agricultural productions to Cd, Zn and As was 48.9%, 33.6% and 30.8%, respectively. Pb and Hg are primarily influenced by transportation and industrial emissions, and their contribution rates to Pb and Hg are 62.9% and 21.2%, respectively. The average value of the I_geo_ and CF that the topsoil is pollution-free overall, but the accumulation of Cd and Hg in local areas is high, and there is a locally moderate level of contamination of Cd, Co and Hg. Cd displayed the greatest potential for environmental pollution, as it possessed the highest the values of CF and I_geo_. Moreover, these discoveries can advise administrative offices in their endeavors to execute focused control methodologies for anticipating the advance spread of heavy metals. Although the quantitative distribution of soil heavy metals can be performed using the PMF model and PCA model, we suggest combining isotope tracing techniques with the PMF model in future studies to conduct regular temporal and spatial sampling of potential contamination sources to enhance the accuracy of the distribution of soil heavy metal sources.

## Figures and Tables

**Table 1:** Detection limit requirement of each index analysis method (measurement unit: mg/kg).

	As	Cd	Co	Cr	Hg	Mn	Ni	Pb	Zn
Detection Limits (DL)	0.3	0.03	1	3	0.0005	10	1	2	2

**Table 2:** Geo-accumulation index.

No. Class	I_geo_ Value	Contamination Level
0	I_geo_ ≤ 0	Practically uncontaminated
1	0 < I_geo_ < 1	Uncontaminated to moderately contaminated
2	1 < I_geo_ < 2	Moderately contaminated
3	2 < I_geo_ < 3	Moderately to strongly contaminated
4	3 < I_geo_ < 4	Strongly contaminated
5	4 < I_geo_ < 5	Strongly to extremely contaminated
6	5 > I_geo_	Extremely contaminated

**Table 3:** The descriptive statistical parameters of heavy metals concentrations in soils (mg/kg).

	As	Cd	Co	Cr	Mn	Ni	Zn	Pb	Hg
N(a)	639	639	639	639	639	639	639	639	639
Mean	14.45	0.21	18.69	81.69	898.41	39.37	79.50	28.11	0.04
Median	14.6	0.186	17.9	81.9	873	39.3	77.1	27.8	0.033
Maximum	38.9	1.153	52.9	202	1783	83.2	237	107	0.662
Minimum	5.2	0.099	5.9	20.5	367	9.6	40.1	14.2	0.015
Standard deviation	4.5	0.099	5.6	17.3	205	9.1	17.2	6	0.047
CV (%)	31	48	30	21	23	23	22	21	113
Background values of Henan Province	11.4	0.074	10	63.8	579	26.7	60.1	19.6	0.034

**Table 4:** Results of PCA of nine heavy metals concentrations in topsoil.

	F1	F2	F3
As	0.49	0.39	0.47
Cd	0.28	0.77	−0.16
Co	0.86	−0.31	−0.02
Cr	0.83	−0.1	−0.02
Mn	0.79	−0.27	0.21
Ni	0.94	−0.02	0.05
Pb	−0.15	0.46	0.69
Zn	0.63	0.36	−0.43
Hg	0.04	0.42	−0.43

## Data Availability

No new data were created or analyzed in this study. Data sharing is not applicable to this article.
